# Pan-Genomic Characterization of the Nudix Hydrolase Gene Family and Its Response to Different Pathogen Strains in Maize (*Zea mays*)

**DOI:** 10.3390/genes17091005

**Published:** 2026-08-26

**Authors:** Shuyao Tang, Yuhan Zheng, Xi Chen, Shuting Zhang, Min Li, Wei Wang, Maqsood Khan, Xiao Xiao, Zhi Nie, Boyi Pi, Siwei Zhang

**Affiliations:** 1 College of Life Sciences, Sichuan Normal University, Chengdu 610011, China; tangshuyao6699@163.com (S.T.);; 2 State Key Laboratory of Tropical Crop Breeding, Sanya Institute of Breeding and Multiplication, School of Tropical Agriculture and Forestry, Hainan University, Sanya 572000, China; 3 General Management Committee of the Suining Campus, Sichuan Normal University, Suining 629000, China; 4 Institute of Biotechnology and Nuclear Technology, Sichuan Academy of Agricultural Science, Chengdu 610011, China

**Keywords:** *Zea mays*, pan-genome, Nudix hydrolase, gene family, stress response

## Abstract

Background: Nudix hydrolases (NHs), characterized by the conserved Nudix motif (GX_5_EX_7_REUXEEXGU), hydrolyze nucleoside diphosphate derivatives and play vital roles in plant stress responses and cellular homeostasis. However, the NH gene family has not been systematically characterized in maize (*Zea mays* L.). Methods: We performed a genome-wide analysis of *ZmNH* (Nudix hydrolase) genes using a maize pan-genome encompassing 26 high-quality genomes. Results: A total of 73 *ZmNH* members were identified, comprising 22 core genes (present in all 26 lines), 28 dispensable genes (present in 2–25 lines), and 23 private genes (present in only one line). Phylogenetic analysis clustered them into three subgroups (Type-I, -II, and -III), with Type-III being the largest (28 members). Gene structure and motif analyses revealed conserved exon–intron architectures and 10 conserved motifs. Nonsynonymous/synonymous substitution ratio (Ka/Ks) calculations across the 26 inbred lines indicated that most *ZmNH* genes were under purifying selection (Ka/Ks < 1), whereas *ZmNH2* and *ZmNH12* showed evidence of positive selection in some lines. Expression profiling under four stress conditions—*Fusarium graminearum*, *Cercospora zeina*, *Exserohilum turcicum*, and *Ostrinia furnacalis*—identified 20 differentially expressed *ZmNH* genes. Notably, *ZmNH9* was consistently responsive across multiple pathogen treatments, suggesting a potential role in broad-spectrum disease resistance. Conclusions: These findings provide a foundation for future functional studies of the maize NH gene family.

## 1. Introduction

Maize (*Zea mays* L.) is one of the most important cereal crops cultivated globally, serving as a critical source of food, livestock feed, and bioenergy [1]. Its remarkable adaptability to diverse agroecological conditions including tolerance to drought, cold, and nutrient-deficient soils has enabled its widespread cultivation across varied climates [2,3]. Nevertheless, abiotic and biotic stresses remain major constraints on maize productivity, resulting in estimated annual yield losses of 20–30% [4,5]. Although the maize genome has been fully sequenced, functional annotations of more than half of the predicted genes still need to be identified, particularly within the gene families associated with stress response and adaptation [6,7].

The Nudix hydrolase (NH) family, characterized by a highly conserved Nudix motif (GX_5_EX_7_REUXEEXGU), catalyzes the hydrolysis of a wide range of nucleoside diphosphate-linked compounds [8]. Members of this superfamily are widely distributed across bacteria, fungi, animals and plants [9], and the conserved Nudix motif forms the catalytic center of these enzymes and is essential for binding divalent metal ions required for substrate hydrolysis [9,10]. This diverse superfamily includes enzymes with specificity for various substrates, including nucleoside triphosphates, dinucleoside polyphosphates, nucleotide sugars, and NADH [11]. Nudix hydrolases play essential roles in maintaining cellular homeostasis by detoxifying reactive metabolites, modulating signaling nucleotides, and alleviating oxidative stress. In plants, these enzymes have been demonstrated to participate in multiple physiological processes, including seed germination [12], root development [13], and stress responses [14].

With the advancement in the field of plant genomics and transcriptomics, an increasing number of NH family members have been identified in different plant species. *Arabidopsis thaliana* possesses 29 Nudix hydrolases (*AtNUDXs*), some of which have been extensively characterized. For instance, *AtNUDX7* and *AtNUDX6* exhibit NADH pyrophosphohydrolase activities and contribute to the modulation of various defense responses [15,16]. Overexpression of *AtNUDX2* confers enhanced tolerance to oxidative stress by maintaining NAD^+^ and ATP levels through nucleotide recycling from free ADP-ribose [17]. Recent studies have also revealed that Nudix hydrolases play important roles in regulating plant hormone signaling and different developmental processes [18]. Phylogenetic studies indicated that plant Nudix hydrolases have undergone lineage-specific expansion, likely driven by environmental adaptation, which suggests their involvement in abiotic stress responses and developmental regulation [19]. NH family members have been identified in different crops. For example, *OsNUDX2* was shown to hydrolyze oxidized nucleotides such as 8-oxo-dGTP and contribute to the detoxification of reactive oxygen species (ROS) induced nucleotide damage under UV stress conditions [20]. *OsNUDX14* affected grain chalkiness in rice and *OsNUDX14*-Cas9 (*nudx14*) induced early flowering and a larger flag leaf angle during the reproductive stage [18]. Genome-wide and molecular characterization studies in barley further revealed the structural diversity and evolutionary conservation of plant NH genes [21]. Compared to *Arabidopsis* and other cultivated crops, relatively little information is available about the NH gene family in maize. Recently, Hufford et al. (2021) [6] published a maize pan-genome comprising 26 high-quality genomes, which captures extensive presence-absence variation (PAV) and structural variation (SV). This landmark study provides an unprecedented resource for comprehensive gene family and functional studies in maize. Subsequent studies have utilized this pan-genome resource to investigate various gene families, including GATA transcription factors [22], PAL genes [23] and terpene synthases [24]. These analyses demonstrated the importance of pan-genome-based approaches for understanding gene family evolution and functional diversification.

Although several maize genes potentially encoding NH proteins have been annotated in genomic databases, systematic analyses of their evolutionary relationships, gene structures, conserved motifs, chromosomal distribution, and expression patterns are still lacking. Moreover, although the NH gene has been extensively characterized as an important regulator of plant growth and stress adaptation in *Arabidopsis* and other plant species, the NH gene family in maize has not yet been systematically identified or comprehensively analyzed [20,25]. In this study, we utilized the maize pan-genome comprising 26 high-quality genomes published by Hufford et al. (2021) [6]. We identified and classified NH gene family members in maize; analyzed their phylogenetic relationships, gene structure, and conserved motifs; and evaluated selection pressures acting on NH genes across different maize varieties. Further, the expression profiles of NH genes under various stress conditions were investigated. This study provides the first systematic analysis of the NH gene family in maize and offers valuable insights for future functional characterization.

## 2. Results

### 2.1. Pan-Genome-Wide Identification of ZmNH Genes

A comprehensive search of the 26 maize pan-genome sequences identified 73 NH gene family members [6]. Based on their presence-absence patterns across the 26 lines, these genes were classified into three categories: (1) core genes (*n* = 22), present in all 26 maize lines; (2) dispensable genes (*n* = 28), present in 2–25 lines; and (3) private genes (*n* = 23), present in only one line (Supplementary Table S1). This distribution is consistent with previous reports on other gene families in the maize pan-genome, suggesting that presence-absence variation (PAV) is a common feature of maize gene families.

### 2.2. Phylogenetic Classification of ZmNH Genes

**Figure 1:**
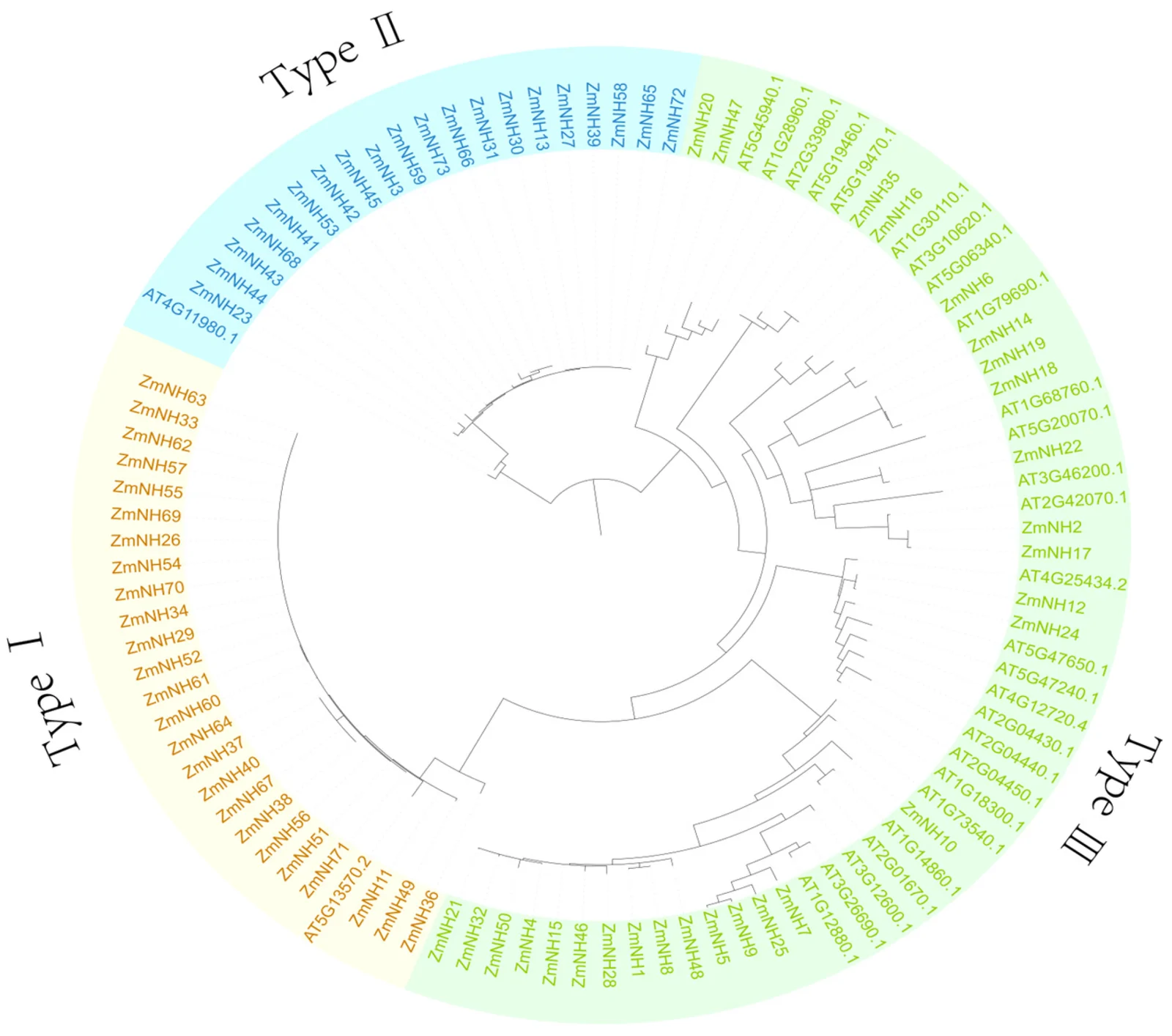
The phylogenetic tree of NH family genes in maize and *Arabidopsis.* Phylogenetic analysis of *ZmNH* and *AtNH* gene families. Maximum likelihood (ML) phylogenetic tree constructed using 73 *ZmNH* (maize) and 29 *AtNH* (*Arabidopsis thaliana*) sequences. The phylogenetic tree reveals three distinct subgroups (Type-I, Type-II, and Type-III), with Type-III being the largest subgroup containing 28 members. The classification is consistent with the previously established criteria for *Arabidopsis* NH proteins.

To investigate the evolutionary relationships and functional diversification of *ZmNH* genes, a maximum likelihood phylogenetic tree was constructed using 73 *ZmNH* and 29 *AtNH* protein sequences. Based on the phylogenetic topology and classification criteria previously established for *Arabidopsis* NH proteins [19], the *ZmNH* genes were divided into three subgroups: Type-I (*n* = 25), Type-II (*n* = 20), and Type-III (*n* = 28) (Figure 1, Supplementary Table S2). Type-III was the largest subgroup, consistent with observations in other plant species. Interestingly, among the 22 core genes, 20 belong to Type-III, indicating high genetic stability of this subgroup. In contrast, Type-I and Type-II contained only 1 core gene each, with the majority being dispensable or private genes.

### 2.3. Conserved Motif and Gene Structure Analysis

**Figure 2:**
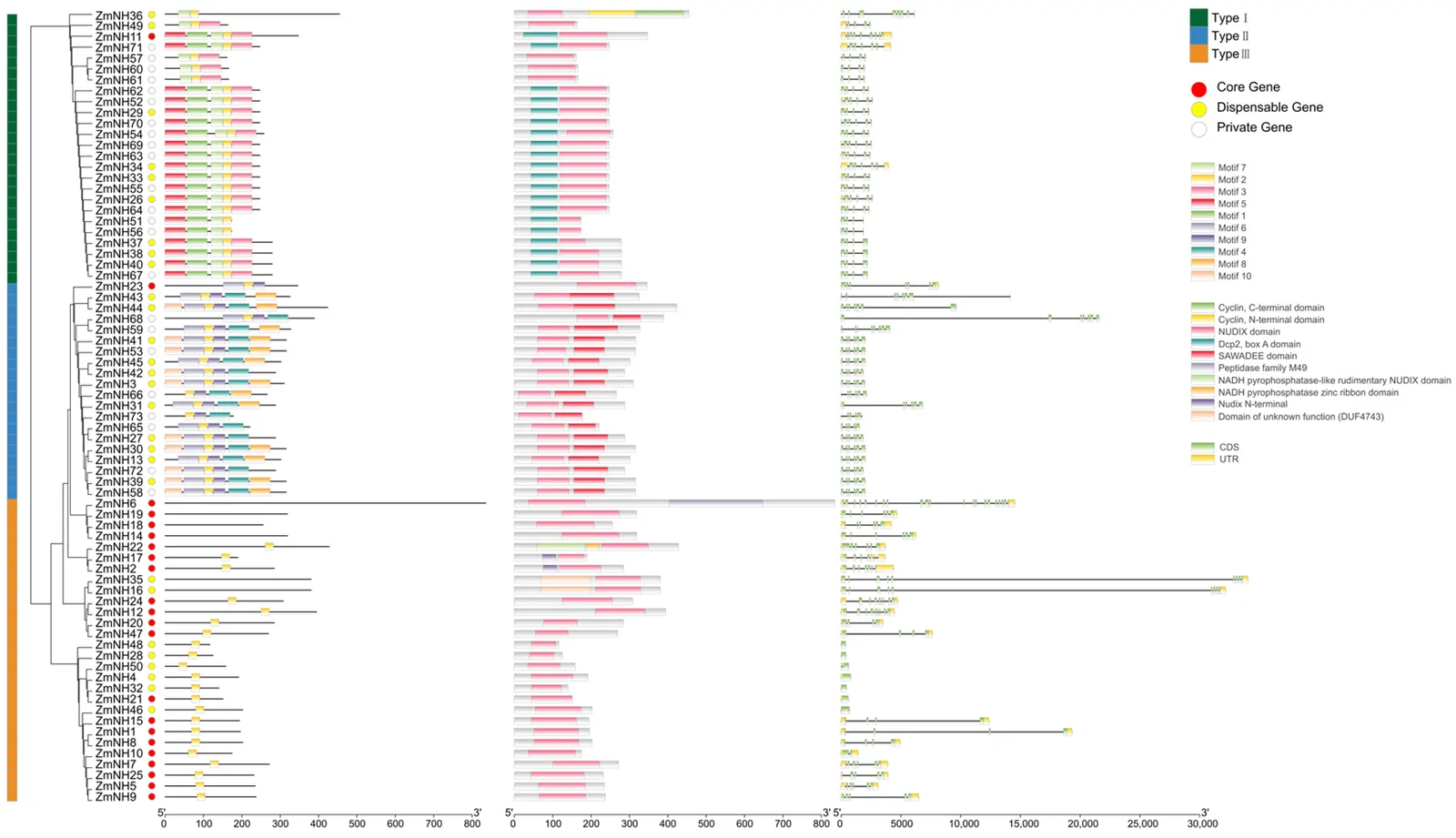
Gene structure and protein domain analyses of *ZmNH* gene family genes in maize. The phylogenetic tree (**left**) shows the classification of 73 *ZmNH* genes into three subgroups (Type-I, Type-II, and Type-III), with gene status indicated as core (present in all 26 lines), dispensable (present in 2–25 lines), or private (present in only one line). Conserved motifs (**middle**) identified by MEME analysis are shown as colored boxes; Motif 2 corresponds to the core NUDIX domain (PF00293), Motif 5 is specific to Type-I proteins and contains the Dcp2 box A domain, and Motif 4 is specific to Type-II proteins and contains the SAWADEE domain. Exon–intron structures (**right**) are displayed with coding sequences (CDS) and untranslated regions (UTR); gene lengths are proportional to the scale bar.

Ten conserved motifs were identified using MEME analysis. Motif 2, corresponding to the core NUDIX domain (PF00293), was present in almost all *ZmNH* proteins (72/73), confirming the reliability of our identification. Notably, Motif 5 was specifically enriched in Type-I proteins and contained residues corresponding to the Dcp2 box A domain, which is involved in mRNA decapping processes. Motif 4 was specifically enriched in Type-II proteins and contained the SAWADEE domain, which is associated with chromatin modification or transcriptional regulation. These domain compositions support the hypothesis that different NH subtypes have evolved distinct functional specializations (Figure 2). Analysis of exon–intron structures revealed significant variation in gene length and intron number among *ZmNH* genes, ranging from 1 to 15 exons (Figure 2).

### 2.4. ZmNH Is Subjected to Different Selection Pressures Among Maize Inbred Lines

**Figure 3:**
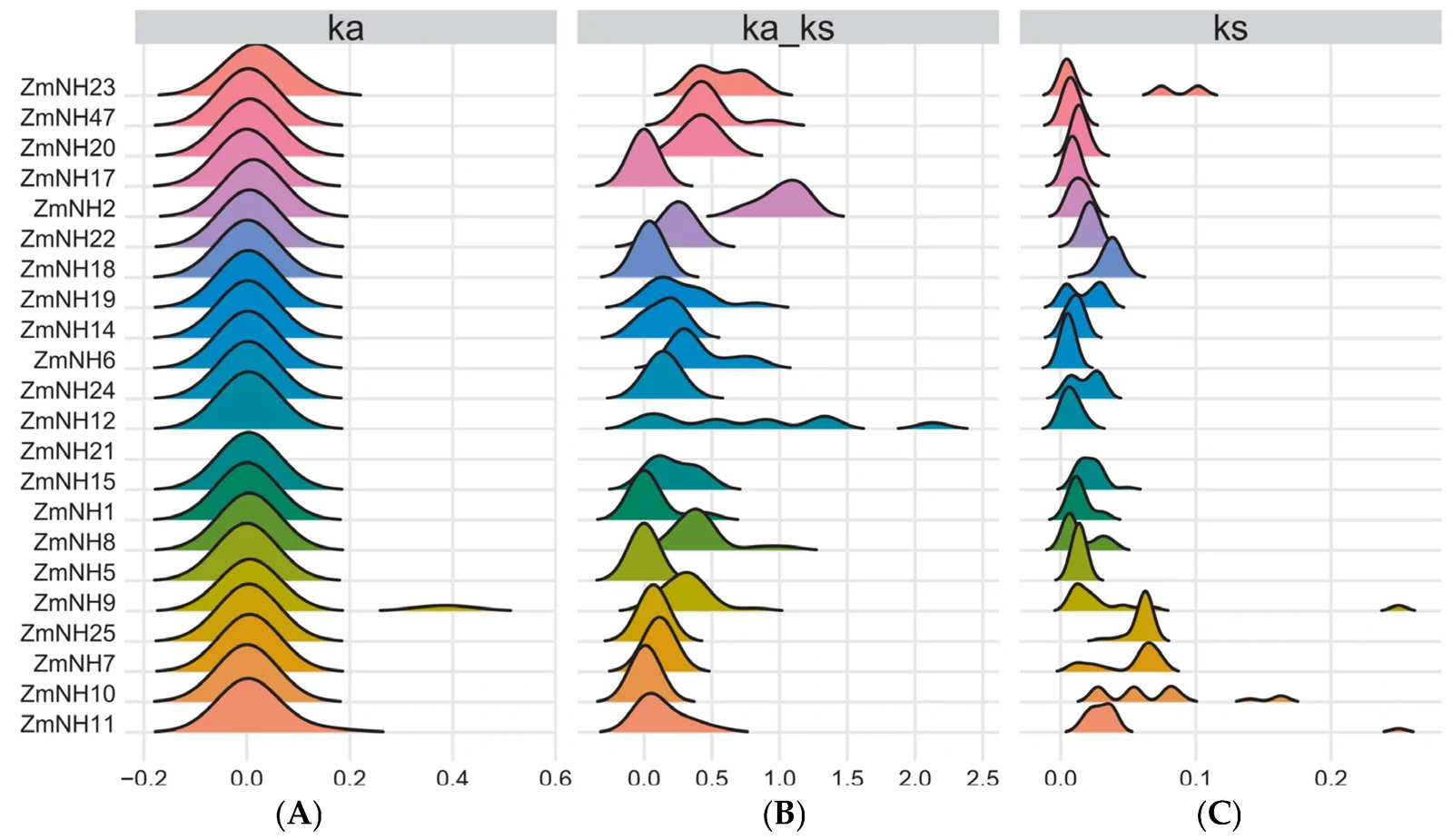
The distribution of Ka, Ks, and Ka/Ks for the core genes of the *ZmNH* gene family in maize. Distribution of (**A**) Ka (nonsynonymous substitution rate), (**B**) Ka/Ks ratio, and (**C**) Ks (synonymous substitution rate) for 22 core *ZmNH* genes.

To investigate the evolutionary forces shaping the *ZmNH* gene family, we calculated Ka, Ks, and Ka/Ks values for orthologous gene pairs across 26 maize inbred lines (Figure 3, Supplementary Table S3). Results revealed that the majority of *ZmNH* genes have Ka/Ks values less than 1 (mean = 0.35), indicating strong purifying selection acting on these genes during maize domestication and improvement. However, notable exceptions were observed: *ZmNH2* and *ZmNH12* showed higher proportions of Ka/Ks > 1 across multiple varieties, suggesting that these genes may have undergone positive selection. Structural variation (SV) analysis revealed that some accessions contained SV events that disrupted the NUDIX domain integrity, potentially affecting gene function.

### 2.5. Expression Profiling of ZmNH Genes Under Pathogen Stress

**Figure 4:**
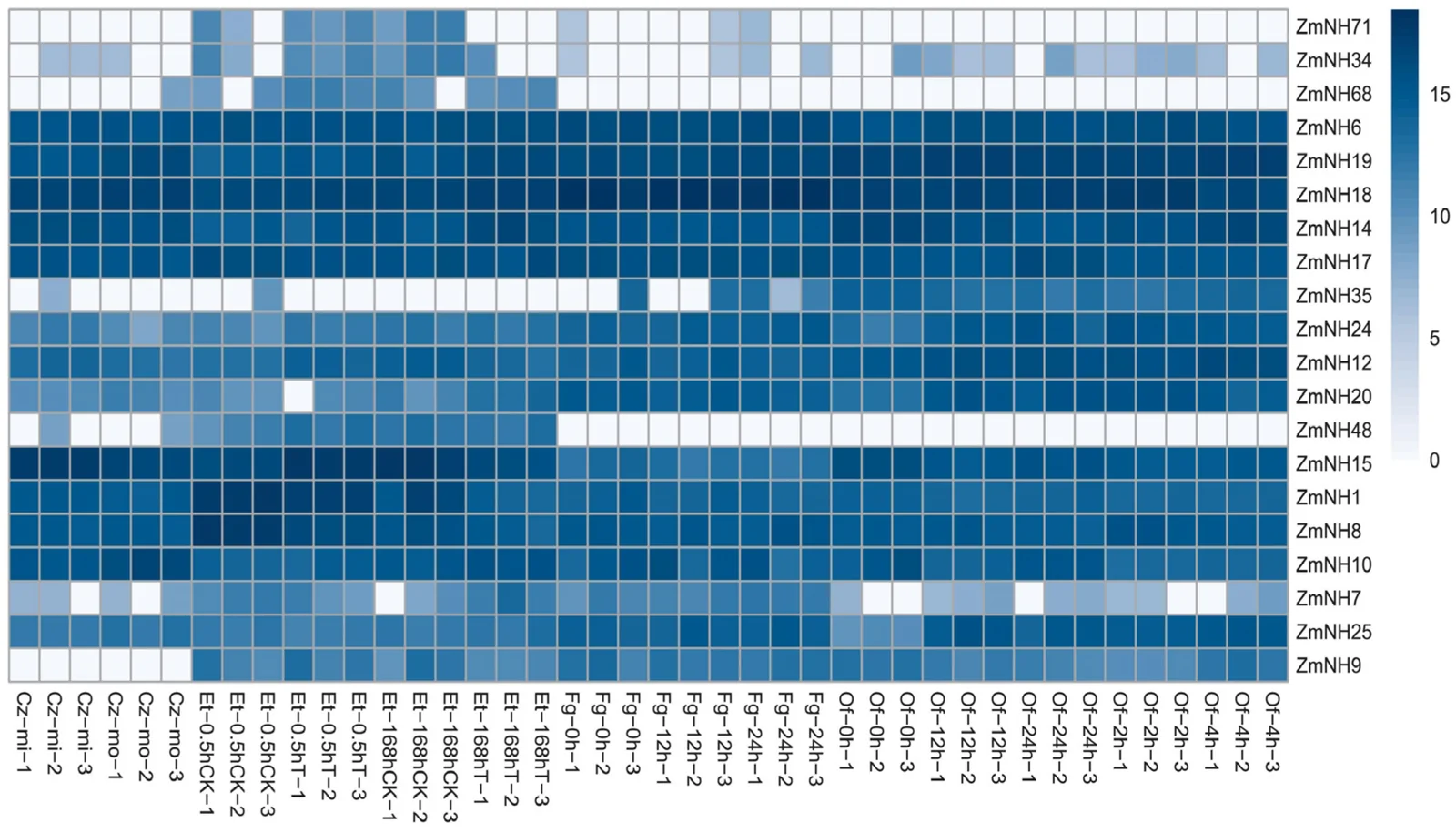
The 20 differentially expressed genes (DEGs) under four biotic stresses. Expression levels are shown as log_2_-transformed transcripts per million (TPM) values.

To investigate the potential functions of *ZmNH* genes in stress responses, we independently performed a bioinformatic re-analysis of publicly available RNA-seq datasets to characterize their expression patterns under inoculation with four different pathogens: *Fusarium graminearum* (Fg), *Cercospora zeina* (Cz), *Exserohilum turcicum* (Et), and *Ostrinia furnacalis* (Of) infestation (Supplementary Figure S1). A total of 20 differentially expressed genes (DEGs) were identified across all treatments (Figure 4). Among these, 12 *ZmNH* genes showed consistent differential expression patterns across multiple treatments, while 8 genes showed treatment-specific responses. Notably, *ZmNH9* showed differential expression in all treatments except Cz infection, suggesting its potential role as a broad-spectrum disease resistance regulator. *ZmNH48* showed specific differential expression under Et infection, indicating that it plays a special role in Et resistance.

**Figure 5:**
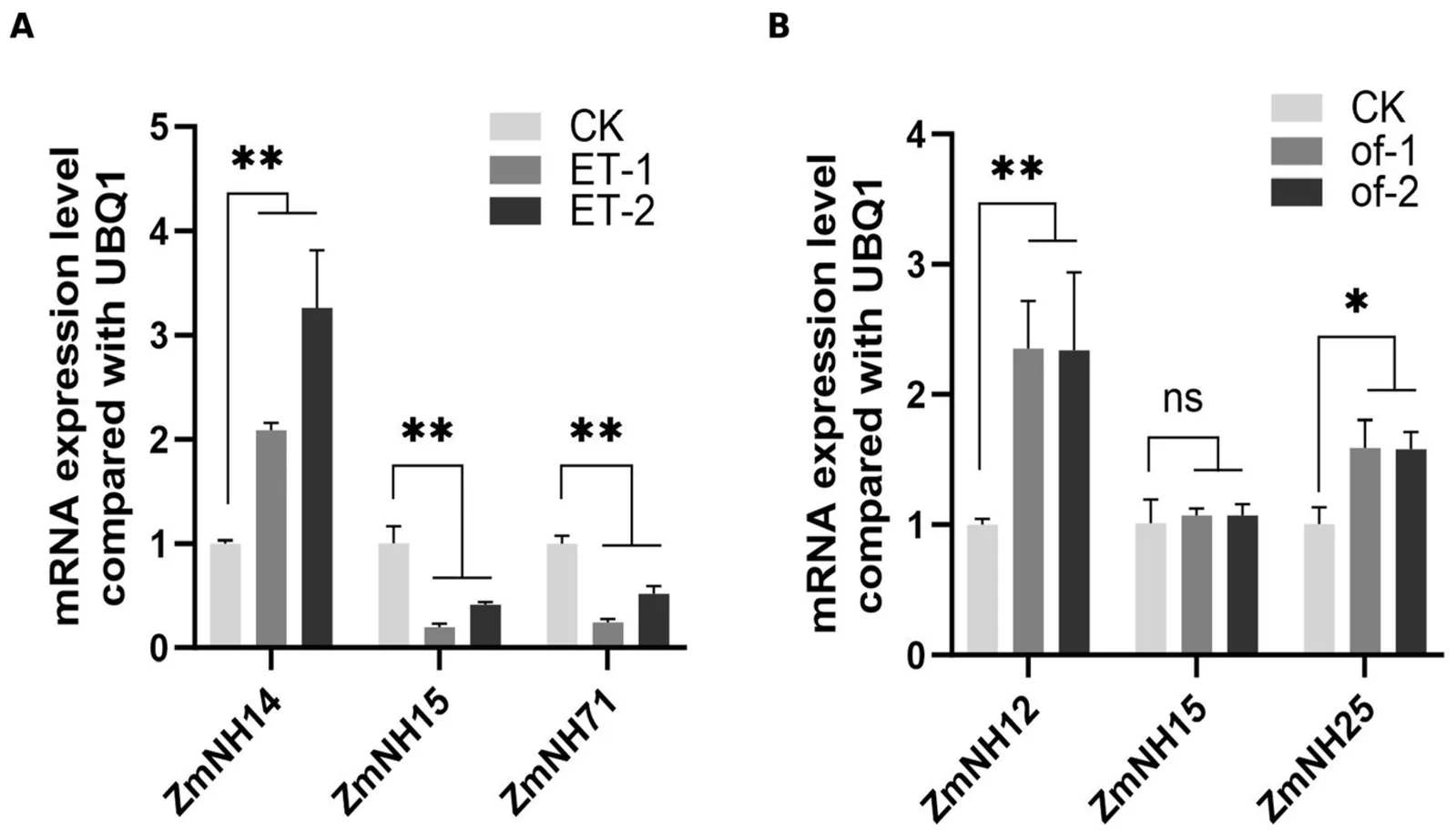
RT-qPCR validation of selected *ZmNH* genes under biotic stress. Relative expression levels of *ZmNH12*, *ZmNH14*, *ZmNH15*, *ZmNH25*, and *ZmNH71* under (**A**) *Exserohilum turcicum* (Et) infection at 168 h post-inoculation (hpi) and (**B**) *Ostrinia furnacalis* (Of) infestation at 24 hpi, compared with mock controls (CK). The maize ubiquitin gene *ZmUBQ1* was used as the internal reference, and relative expression was calculated using the 2^−ΔΔCt^ method. Error bars represent standard deviation from three biological replicates. Asterisks indicate significant differences (* *p* < 0.05, ** *p* < 0.01; ns, not significant; Student’s *t*-test).

To validate the RNA-seq expression patterns, reverse transcription quantitative PCR (RT-qPCR) was performed on five selected *ZmNH* genes (*ZmNH12*, *ZmNH14*, *ZmNH15*, *ZmNH25*, and *ZmNH71*) using independently prepared biological samples. Under Et infection, *ZmNH14* was significantly upregulated, while *ZmNH15* and *ZmNH71* were significantly downregulated, consistent with the transcriptome analysis. Under Of infestation, *ZmNH12* and *ZmNH25* were significantly upregulated, whereas *ZmNH15* showed no significant change, which also agreed with the RNA-seq data. Overall, the RT-qPCR results were in good agreement with the bioinformatic re-analysis, confirming the reliability of the expression profiling (Figure 5).

### 2.6. Expression Profiling Across Maize Inbred Lines

**Figure 6:**
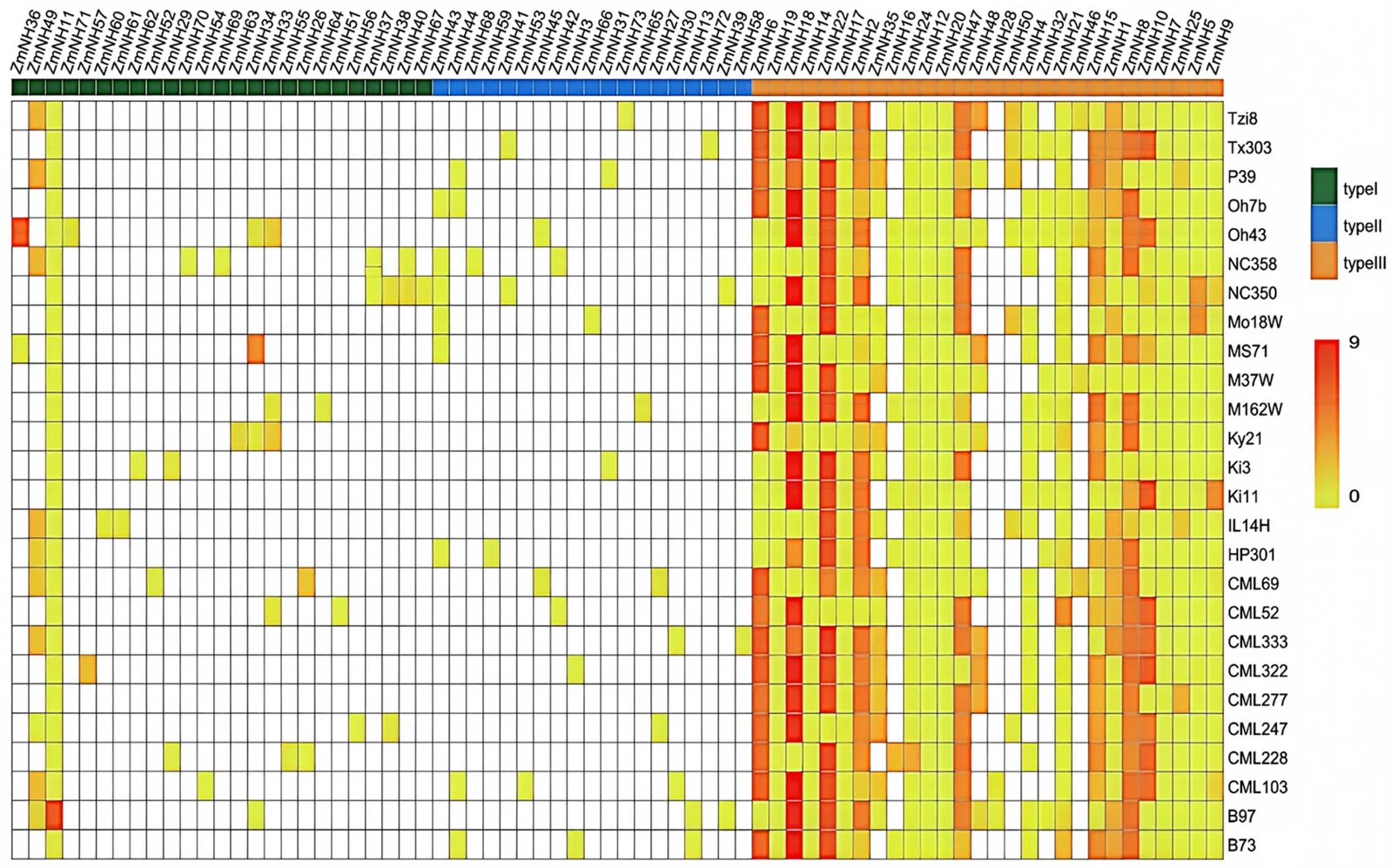
The expression levels of the *ZmNH* gene family in 26 maize inbred lines. Heatmap showing the expression levels (log_2_-transformed TPM) of 73 *ZmNH* genes across 26 diverse maize inbred lines representing the maize pan-genome. Genes are clustered by phylogenetic subtypes (Type-I, Type-II, Type-III). The color gradient indicates expression levels from low (blue) to high (red). Type-III genes generally show higher expression frequency and abundance compared to Type-I and Type-II genes. *ZmNH* genes with constitutive expression across all lines include *ZmNH6*, *ZmNH18*, *ZmNH22*, *ZmNH2*, *ZmNH15*, and *ZmNH8*.

Analysis of expression data across different maize inbred lines revealed that Type-III genes had the highest expression frequency and abundance, consistent with their classification as core genes. Twenty-one *ZmNH* genes showed constitutive expression across all tested inbred lines (Figure 6). Among these, *ZmNH6*, *ZmNH18*, *ZmNH22*, *ZmNH2*, *ZmNH15*, and *ZmNH8* showed relatively high expression levels in most lines, suggesting that they may play essential roles in growth and development of maize.

## 3. Discussion

This study systematically identifies the *ZmNH* gene family using a pan-genome framework built from 26 high-quality maize genomes. It comprehensively analyzes the gene family’s diversity and functional potential in three dimensions: evolutionary development, structural characteristics, and expression pattern/regulation.

### 3.1. Pan-Genome-Wide Analyses of the NH Gene Family Revealed Three Subtypes

Based on 26 high-quality genomes, this study identified 73 members of the NH gene family in the maize pan-genome system. This indicates the substantial genetic diversity and complex structural composition of this gene family in maize. These genes were classified into three categories: Type-I (25), Type-II (20), and Type-III (28), with Type-III containing the most core genes (20) and exhibiting high genetic stability. This suggests its pivotal role in basic physiological processes. In contrast, Type-I and Type-II each contain only one core gene, with most members being dispensable or private genes, exhibiting stronger presence-absence variation (PAV), which may reflect their potential contribution to adaptation to specific environments or cultivar-specific traits. This distribution pattern aligns with the pan-genome theory framework, where core genes maintain basic functions, and variable genes participate in adaptive evolution [26]. Overall, this classification reflects the evolutionary divergence of *ZmNH* genes, with each type representing distinct phylogenetic lineages that originated from ancient duplication events, and implies that their functions may exhibit subtype specificity. Notably, each type exhibits conserved sequence motifs and specific evolutionary conservation patterns across species, further supporting the functional and evolutionary distinctness of these subtypes.

Notably, the vast majority of core genes (20/22) are assigned to Type-III, while most private genes (present in only one strain) are scattered across different subtypes. These findings indicate that some *ZmNH* genes may function adaptively in particular genetic backgrounds. For instance, *ZmNH36* is predominantly found in the Ms71 and Oh43 strains, suggesting its potential involvement in regulating traits unique to these lines, such as physiological or stress resistance characteristics. Furthermore, the presence of 28 dispensable genes indicates widespread “presence-absence variation” (PAV) within the *ZmNH* gene family at the population level, which may result from functional redundancy or environmental adaptation mechanisms retained during natural selection or artificial domestication [27,28].

By comparing the phylogenetic relationships with the *Arabidopsis* NH gene family, we hypothesize that the classification into these three types reflects a conserved evolutionary foundation. Previous studies have shown that plant Nudix hydrolases have undergone lineage-specific expansion events, particularly exhibiting notable adaptive evolutionary traits in response to abiotic stresses [19]. Thus, the differentiation of the three *ZmNH* types in maize is likely closely associated with their diversified roles in signal transduction, metabolic regulation, and stress responses.

### 3.2. Evolutionary History of NH Gene Family

*(1)*  *Gene Structure Analysis*

Gene structure and conserved motif analysis further revealed the functional diversity and evolutionary conservation within the *ZmNH* gene family. We identified 10 conserved functional domain motifs in the 73 *ZmNH*-encoded proteins, with Motif 2 present in almost all members. This motif corresponds to the typical NUDIX domain (GX_5_EX_7_REUXEEXGU), which is the core region for catalytic activity, playing a role in substrate binding and metal ion coordination. The high conservation of this domain indicates its irreplaceable key role in maintaining cellular homeostasis.

More notably, different types of *ZmNH* carry unique auxiliary motifs: Motif 5 is specifically enriched in Type-I proteins and is associated with the Dcp2 box A domain, indicating that these genes may be involved in the mRNA decapping process or RNA metabolism regulation; whereas Motif 4 is exclusive to Type-II members and is associated with the SAWADEE domain, which has been reported to be involved in chromatin modification or transcriptional regulation. This suggests that, despite overall sequence similarity, there are significant differences in protein functional modules between different subtypes, potentially mediating distinct substrate recognition or interaction networks, leading to subfunctionalization or neofunctionalization. The differences in these domain combinations strongly support the evolutionary trajectory of neofunctionalization or subfunctionalization in the *ZmNH* gene family following gene duplication.

Additionally, exon–intron structure analysis reveals that, despite the overall conservation of gene structure, some private or dispensable genes exhibit abnormal splicing patterns or domain deletions, possibly due to structural variations (SV). Such variations may lead to a loss of protein function or the acquisition of new regulatory abilities, thereby driving phenotypic diversity between species or cultivars.

*(2)*  *ZmNH is Subjected to Different Selection Pressures Among Maize Inbred Lines*

The Nudix hydrolase family demonstrates significant dynamics in evolutionary traits throughout plant evolution. Through the analysis of Ka/Ks ratios of core *ZmNH* genes in 26 maize inbred lines, we found that most genes have a Ka/Ks ratio of < 1, suggesting that they are under strong purifying selection, indicating that their amino acid sequences are highly conserved, and their functions are resistant to change. This helps maintain their fundamental roles in cellular homeostasis regulation, such as eliminating reactive metabolites, regulating signaling nucleotides, and responding to oxidative damage. However, a higher proportion of Ka/Ks > 1 was observed in certain specific genes (such as *ZmNH2*, *ZmNH12*), indicating that these genes may have experienced positive selection during maize domestication or improvement. In conjunction with previous reports on the critical role of *AtNUDX1* in ROS scavenging and DNA repair, we hypothesize that these positively selected *ZmNH* genes may confer new phenotypic plasticity to plants, such as enhanced response to drought, salt stress, or pathogen infection. Further research on their relationship with agronomic traits is warranted.

Simultaneously, structural variation (SV) analysis reveals that SV events in certain strains disrupt the integrity of the NUDIX domain, resulting in the production of atypical gene forms in some accessions. These genetic variations could weaken enzyme activity or potentially provide new regulatory potential by modifying the patterns of expression regulation. Therefore, the evolutionary dynamics of the *ZmNH* gene family are not solely the result of selection at the sequence level, but rather a process jointly driven by genomic structural rearrangements and interactions with the environment.

### 3.3. Expression Profiling of the NH Gene Family Under Different Stresses and Across Different Inbred Lines

Transcriptome data analysis revealed the intricate expression patterns of the *ZmNH* gene family under various biotic stress conditions. A total of 20 differentially expressed genes (DEGs) were identified in plants infested with *Fusarium graminearum* (Fg), *Cercospora zeina* (Cz), *Exserohilum turcicum* (Et) and *Ostrinia furnacalis* (Of), with 12 genes exhibiting distinct stress response specificities across all samples. *ZmNH9* was differentially expressed in all treatments except for Cz infection, indicating that it might function as a broad-spectrum disease resistance regulator, possibly acting as a central regulatory factor in plant defense responses. *ZmNH48* was predominantly expressed in Et-infected samples, suggesting that its function may be confined to specific pathogen recognition or the activation of defense signaling pathways.

More importantly, some genes show strict condition-dependent expression. For instance, *ZmNH21*, *ZmNH48*, and *ZmNH68* are induced exclusively in the Cz and Et samples, exhibiting typical “conditional” activation characteristics, suggesting that they may serve as environmental sensors involved in local immune regulation. A total of 21 *ZmNH* genes exhibit constitutive expression in all samples, likely contributing to basic cellular metabolism and homeostasis maintenance, embodying their “housekeeping gene” characteristics.

Expression profiling across 26 maize inbred lines further revealed the genetic diversity of *ZmNH* expression. Overall, Type-III genes show the highest expression frequency and abundance, with most being core genes, highlighting their universal importance in basic life processes. In contrast, Type-I/II genes generally exhibit lower expression levels, which may indicate regulatory branches with restricted functions or tissue-specific regulation. Specifically, *ZmNH11* is detectable in all lines but is significantly overexpressed in the B97 line (Figure 6), indicating that its expression is regulated by genetic background and may be involved in lineage-specific regulatory networks or variations in cis-regulatory elements. Notably, *ZmNH36* was highly expressed only in the Oh43 line.

To further validate the findings of this study, the candidate genes identified here (e.g., *ZmNH9*, *ZmNH48*, and *ZmNH6*) could be functionally characterized through overexpression and CRISPR/Cas9-mediated knockout, followed by phenotypic assays under pathogen challenge to directly assess their contributions to maize disease resistance. In addition, extending the current pan-genome analysis to a broader set of maize inbred lines and wild relatives would help reveal the evolutionary dynamics and natural allelic variation in the NH gene family, and may facilitate the identification of favorable alleles for breeding.

## 4. Materials and Methods

### 4.1. Identification of Maize NH Gene Family

In this study, we utilized the maize pan-genome resource of 26 maize inbred lines established by Hufford et al. (2021) [6] (Supplementary Table S5). The gene models were generated using the Ensembl Compara pipeline, which clusters gene models through phylogenetic tree construction and sequence similarity analysis to define orthogroups. Allelic variants versus paralogous genes were distinguished through gene tree–species tree reconciliation analysis, ensuring that allelic variants from different lines were correctly grouped as orthologs while paralogous genes arising from duplication events were identified separately. This rigorous curation prevents inflation of gene family size by properly distinguishing orthologs, allelic variants, isoforms, and line-specific duplicates. Protein domains of the representative transcripts encoded by these pan-genome genes were annotated using InterProScan (v5.22-61.0) [29] against the Pfam database [30]. Based on these annotations, genes encoding proteins containing the NUDIX domain (PF00293) were identified as members of the Nudix hydrolase (NH) gene family in maize.

### 4.2. Phylogenetic Analysis and Classification

The 73 identified maize NH genes and 29 *Arabidopsis AtNH* genes were subjected to phylogenetic analysis. Multiple sequence alignment was performed using MAFFT v7.520 [31], and the alignment was trimmed with trimAl v1.4.rev15 (-gappyout -phylip_paml) [32]. The best-fit substitution model was determined by ProtTest v3.4.2 [33]. A maximum-likelihood (ML) tree was then constructed using RAxML v8.2.12 with the PROTGAMMAILGX model (-m PROTGAMMAILGX -N 100 -f a -k -d) [34], and visualized in iTOL (https://itol.embl.de/) [35].

To classify the identified genes, the phylogenetic tree topology was analyzed in conjunction with exon–intron organization and conserved domain architecture. Genes clustering together with consistent gene structure and domain configurations were assigned to the same type (Figure 2). The multiple sequence alignment and ML tree files are provided as Supplementary Files S1–S3.

### 4.3. Analysis of the Gene Structure of NH

Motif prediction was performed on the protein sequences encoded by the representative transcripts of 73 maize NH family genes using MEME [36] (v5.4.1, -maxw 50 -nmotifs 10 -protein), with 10 predicted motifs and a maximum motif width of 50 aa. Domain location information was obtained from Pfam annotation files. Gene structures were obtained from the genome GFF3 annotation files of each strain.

### 4.4. Ka/Ks Calculation

The protein and coding sequence (CDS) sequences of NH genes in 26 maize genomes were obtained from the study by Hufford et al. (2021) [6]. Orthologous sequences of the 22 core *ZmNH* genes across the 26 maize genomes were compared and Ka/Ks values were calculated using WGDI v0.6.5 [37]. The Ridgeline plot of Ka/Ks values was generated using the ggridges and ggplot2 packages in R v4.0.3.

### 4.5. RNA-seq Datasets Collection and Analyses

To explore the expression of *ZmNH* genes under stresses, we independently performed a comprehensive bioinformatic re-analysis of publicly available RNA-seq datasets. All read processing, quantification, and differential expression analyses were conducted using standardized computational pipelines; the results presented in Figure 4 represent original analyses rather than previously published expression values. Raw sequencing reads were obtained from the NCBI Sequence Read Archive (SRA) and uniformly re-processed to generate expression matrices and identify differentially expressed genes. Specifically, a total of 42 RNA-seq samples were retrieved from public databases, including 9 samples from *Fusarium graminearum*-inoculated and uninoculated B73 (NCBI BioProject under accession number PRJNA730310) [38], 6 samples from leaves with minimal and moderate *Cercospora zeina* infection (NCBI BioProject under accession number PRJNA564795) [39], 12 samples from the *Exserohilum turcicum* infection time series experiment using wild-type and *Ht1* transgenic plants (NCBI Gene Expression Omnibus (GEO) under accession number GSE206951) [40], and 15 leaf samples from maize hybrid Jingke968 infested with Asian corn borer (*Ostrinia furnacalis*) (NCBI BioProject under accession number PRJNA772910) [41]. Detailed sample information and SRA accession numbers are provided in Supplementary Table S4.

The gene expression matrix was generated using the abundance_estimates_to_matrix.pl script from Trinity [42] (v2.14.0, parameters: --est_method RSEM --aln_method bowtie, indicating the use of RSEM to calculate transcript expression levels and bowtie for genome alignment). The Trinity (v2.14.0) software package script run_DE_analysis.pl (parameter: --method edgeR) was used for differential gene expression analysis. The analyze_diff_expr.pl script (parameters: -C 1 -P 0.05) was used to screen differentially expressed genes. Finally, we used a custom script to draw a heatmap of the expression levels (log_2_-transformed transcripts per million, log_2_TPM) of differential genes. Heatmaps were generated in R (v4.0.3) using the ComplexHeatmap package.

### 4.6. Plant Materials and Treatments

To validate the expression patterns of selected *ZmNH* genes, RT-qPCR was performed on two sets of biological samples. For the *Exserohilum turcicum* (Et) treatment, maize inbred line B73 was inoculated with *E. turcicum* following the general Northern Leaf Blight (NLB) inoculation approach described by Thatcher et al. (2023) [40]. Leaf tissues were collected at 168 h post-inoculation (hpi), with mock-inoculated plants as controls (CK). For the *Ostrinia furnacalis* (Of) treatment, maize hybrid Jingke968 at the mid-whorl stage (30–35 days after germination) was infested with 20 third-instar *O. furnacalis* larvae per whorl, following Guo et al. (2019) [41]. Leaf tissues were collected at 24 h post-infestation (hpi), with uninfested plants at 0 h as controls (CK). All samples were frozen in liquid nitrogen and stored at −80 °C. Each treatment included three biological replicates.

### 4.7. RNA Extraction and RT-qPCR Analysis

Total RNA was extracted using a polysaccharide and polyphenol plant RNA extraction kit (FOREGENE, RE-05024). Reverse transcription was performed with RT Easy™ II Kit (FOREGENE, RT-01022) using 1000 ng total RNA in 20 μL (42 °C, 15 min; 85 °C, 5 min). RT-qPCR was conducted using SYBR qPCR SuperMix Plus (Novoprotein, E096-01B) on a SLAN-96S system (Shanghai Helical Medical). Each 10 μL reaction contained 5 μL SuperMix (2×), 0.5 μL each primer (10 μM), and 4 μL cDNA. Cycling conditions: 95 °C, 2 min; 39 cycles of 95 °C, 15 s and 58 °C, 30 s. Melting curve analysis (60–95 °C) verified specificity. Primers were designed using Primer5 with amplicons ~100 bp (Supplementary Table S6). Relative expression was calculated by the 2^−ΔΔCt^ method using *ZmUBQ1* as the reference gene.

## 5. Conclusions

This study provides the first comprehensive analysis of the Nudix hydrolase gene family in maize using a pan-genome approach. We identified 73 *ZmNH* genes, classified into three subtypes, and analyzed their evolutionary relationships, gene structures, and expression patterns under various stress conditions. Key findings include: the identification of 22 core genes, 28 dispensable genes, and 23 private genes; the classification of *ZmNH* genes into three subtypes with distinct domain compositions; the detection of purifying selection acting on most genes, with *ZmNH2* and *ZmNH12* showing signs of positive selection; and the identification of stress-responsive genes including *ZmNH9* as a potential broad-spectrum disease resistance regulator.

This pan-genome-wide analysis provides valuable resources and candidate targets for future functional studies. Genes that have undergone positive selection, carry structural variations, and respond significantly to stress (such as *ZmNH9* and *ZmNH48*) should be prioritized for functional validation. Given the multiple roles of Nudix hydrolases in ROS clearance, DNA repair, calcium signaling regulation, and energy metabolism, their potential application in improving crop stress resistance is extensive. Future research should focus on functional validation of key candidate genes through transgenic overexpression/knockout experiments, protein interaction analysis, substrate specificity assessment, and association studies with important agronomic traits.

## Data Availability

All data in this study are available within the paper and within its Supplementary Materials published online.

## Supplementary Materials

The following supporting information can be downloaded at: https://www.mdpi.com/article/10.3390/genes17091005/s1, Table S1: Pan-genome gene information of maize *ZmNH* in 26 maize genomes; Table S2: Classification of 73 members of the *ZmNH* gene family in maize: Table S3: The Ka, Ks, and Ka/Ks values of the core genes of the *ZmNH* family in maize; Table S4: Public availability and sample information of all raw RNA-seq data used in this study; Table S5: Assembly information and GenBank accession numbers of the 26 maize inbred line genomes; Table S6: Primer sequences used for RT-qPCR analysis; Figure S1: The expression patterns under four different pathogen strains: *Cercospora zeina* (Cz), *Exserohilum turcicum* (Et), *Fusarium graminearum* (Fg) and *Ostrinia furnacalis* (Of) feeding; File S1: Multiple sequence alignment of 73 *ZmNH* and 29 *AtNH* protein sequences used for phylogenetic analysis (PHYLIP format). File S2: Maximum likelihood phylogenetic tree with bootstrap support values, labeled with *ZmNH* gene names (Newick format); File S3: Maximum likelihood phylogenetic tree with bootstrap support values, labeled with original Ensembl protein IDs (Newick format).
