# Supplementary material for: Pan-Genomic Characterization of the Nudix Hydrolase Gene Family and Its Response to Different Pathogen Strains in Maize (Zea mays)
Source: Genes (Basel). 2026 Aug 26;17(9):1005. doi: 10.3390/genes17091005 (PMC13606471; doi:10.3390/genes17091005)
Supplement: Supplementary file 1 [file genes-17-01005-s001.zip › Supplementary Figure S1.pdf]

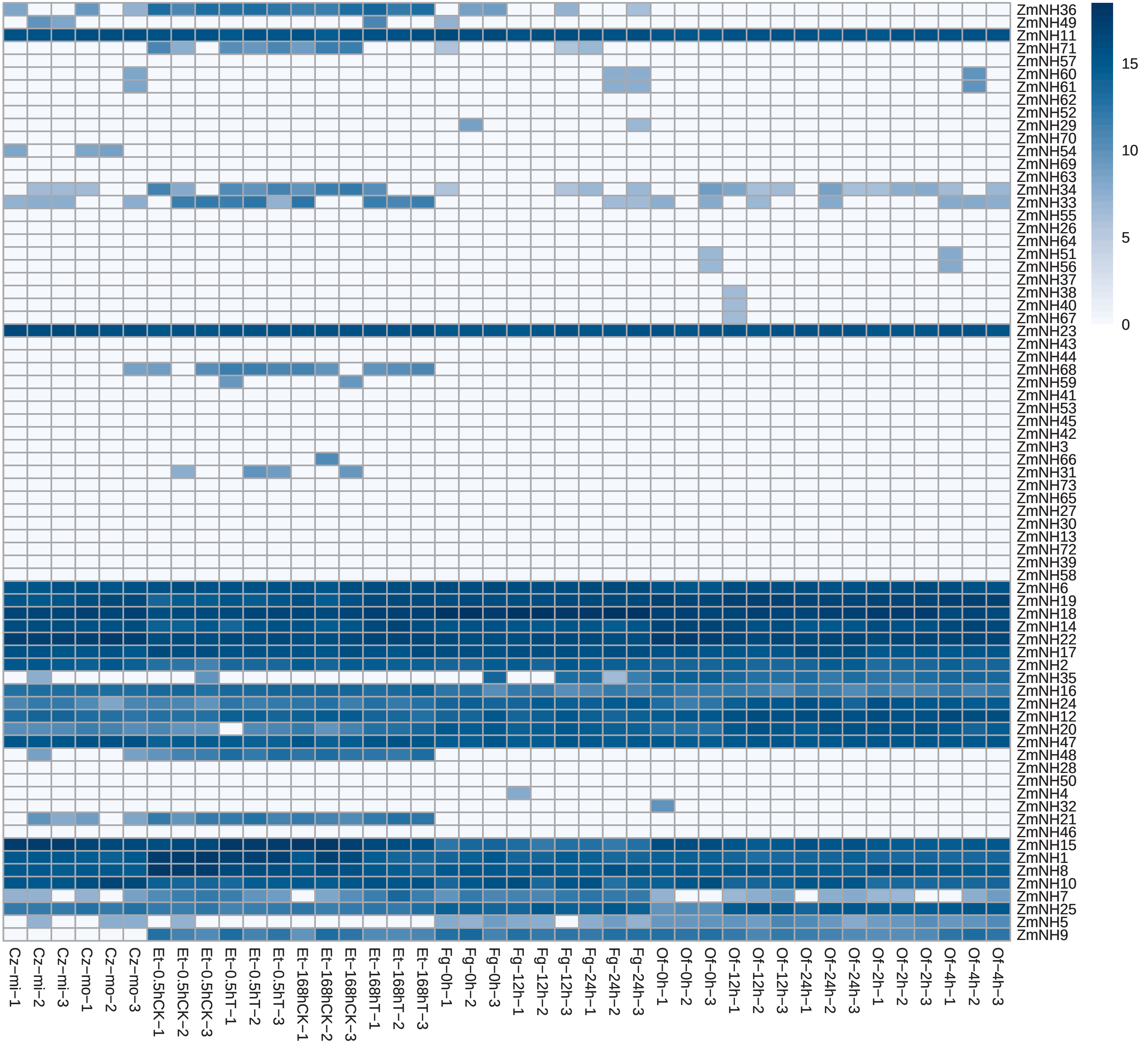
ZmNH36
ZmNH49
ZmNH11
ZmNH71
ZmNH57
ZmNH60
ZmNH61
ZmNH62
ZmNH52
ZmNH29
ZmNH70
ZmNH54
ZmNH69
ZmNH63
ZmNH34
ZmNH33
ZmNH55
ZmNH26
ZmNH64
ZmNH51
ZmNH56
ZmNH37
ZmNH38
ZmNH40
ZmNH67
ZmNH23
ZmNH43
ZmNH44
ZmNH68
ZmNH59
ZmNH41
ZmNH53
ZmNH45
ZmNH42
ZmNH3
ZmNH66
ZmNH31
ZmNH73
ZmNH65
ZmNH27
ZmNH30
ZmNH13
ZmNH72
ZmNH39
ZmNH58
ZmNH6
ZmNH19
ZmNH18
ZmNH14
ZmNH22
ZmNH17
ZmNH2
ZmNH35
ZmNH16
ZmNH24
ZmNH12
ZmNH20
ZmNH47
ZmNH48
ZmNH28
ZmNH50
ZmNH4
ZmNH32
ZmNH21
ZmNH46
ZmNH15
ZmNH1
ZmNH8
ZmNH10
ZmNH7
ZmNH25
ZmNH5
ZmNH9
15
10
5
0
Cz−mi−1
Cz−mi−2
Cz−mi−3
Cz−mo−1
Cz−mo−2
Cz−mo−3
Et−0.5hCK−1
Et−0.5hCK−2
Et−0.5hCK−3
Et−0.5hT−1
Et−0.5hT−2
Et−0.5hT−3
Et−168hCK−1
Et−168hCK−2
Et−168hCK−3
Et−168hT−1
Et−168hT−2
Et−168hT−3
Fg−0h−1
Fg−0h−2
Fg−0h−3
Fg−12h−1
Fg−12h−2
Fg−12h−3
Fg−24h−1
Fg−24h−2
Fg−24h−3
Of−0h−1
Of−0h−2
Of−0h−3
Of−12h−1
Of−12h−2
Of−12h−3
Of−24h−1
Of−24h−2
Of−24h−3
Of−2h−1
Of−2h−2
Of−2h−3
Of−4h−1
Of−4h−2
Of−4h−3
